# Attitudes and practices of public health academics towards research funding from for-profit organizations: cross-sectional survey

**DOI:** 10.1007/s00038-020-01416-0

**Published:** 2020-08-25

**Authors:** Rima Nakkash, Ahmed Ali, Hala Alaouie, Khalil Asmar, Norbert Hirschhorn, Sanaa Mugharbil, Iman Nuwayhid, Leslie London, Amina Saban, Sabina Faiz Rashid, Md Koushik Ahmed, Cecile Knai, Charlotte Bigland, Rima A. Afifi

**Affiliations:** 1 Faculty of Health Sciences, American University of Beirut, Beirut, Lebanon; 2 Independent Consultant, Minneapolis, MN USA; 3 School of Public Health and Family Medicine, University of Cape Town, Cape Town, South Africa; 4 James P. Grant School of Public Health, BRAC University, Dhaka, Bangladesh; 5 London School of Hygiene and Tropical Medicine, London, UK; 6 UK Specialty Registrar, Severn Postgraduate Medical Education School of Public Health, Health Education England, London, UK; 7 Department of Community and Behavioral Health, College of Public Health, University of Iowa, 145 N Riverside Drive, Iowa City, IA USA

**Keywords:** For-profit corporation, Public health, Funding, Conflict of interest, Commercial determinants of health, Unhealthy commodity industries

## Abstract

**Objectives:**

The growing trend of for-profit organization (FPO)-funded university research is concerning because resultant potential conflicts of interest might lead to biases in methods, results, and interpretation. For public health academic programmes, receiving funds from FPOs whose products have negative health implications may be particularly problematic.

**Methods:**

A cross-sectional survey assessed attitudes and practices of public health academics towards accepting funding from FPOs. The sampling frame included universities in five world regions offering a graduate degree in public health; 166 academics responded. Descriptive, bivariate, and logistic regression analyses were conducted.

**Results:**

Over half of respondents were in favour of accepting funding from FPOs; attitudes differed by world region and gender but not by rank, contract status, % salary offset required, primary identity, or exposure to an ethics course. In the last 5 years, almost 20% of respondents had received funding from a FPO. Sixty per cent of respondents agreed that there was potential for bias in seven aspects of the research process, when funds were from FPOs.

**Conclusions:**

Globally, public health academics should increase dialogue around the potential harms of research and practice funded by FPOs.

**Electronic supplementary material:**

The online version of this article (10.1007/s00038-020-01416-0) contains supplementary material, which is available to authorized users.

## Introduction

For-profit organization (FPO)-sponsored academic education, research, and practice have increased recently in parallel with dwindling alternative sources of funding, or because they are perceived to permit more innovation and translation (Nestle 2016; Fabbri et al. 2018). We use the term FPO to mean any national or international organization that sells consumer products related to food and beverages, tobacco, alcohol, and other organizations like pharmaceutical, gambling, arms dealing or manufacturing, health insurance companies, and the petroleum industry. The collaboration between research, science, academics, and FPOs has recently been a widely debated topic globally (Readon 2018; Marks 2019; Marten and Hawkins 2018; Dyer 2020).

The growing trend of FPO-funded university research has led to concerns about potential COI and biases in research methods, results, and interpretation. Industry-funded research tends to produce results favouring the benefits or reducing the harm of the sponsor’s products, as opposed to results produced by independent research (Nestle 2016; Bero et al. 2007; Lundh et al. 2017; Moynihan et al. 2019; Babor and Robaina 2013). FPOs have manipulated the research process on multiple levels including the design, results, and publication (Brandt 2012). Scoping and systematic reviews examining the influence of industry funding have found interference in the research agenda, the selection and framing of research priorities, and research questions, and in design and data analysis (Fabbri et al. 2018; McCambridge and Mialon 2018; Rasmussen et al. 2018). In response, Cochrane has recently proposed a more rigorous conflict of interest (COI) policy (Lundh et al. 2017; Soares-Weiser 2019). Despite this overwhelming evidence of industry interference in research, interactions between health researchers and FPOs remains common and often hidden. Recent scholarship also suggests that mere disclosures of COI should not be the priority for governing interactions, rather ‘sequestration’—elimination of the relationship, is necessary (Goldberg 2019).

Behaviours are influenced by attitudes. Despite different levels of awareness of the tactics of different industries (Collin et al. 2017), health stakeholders generally acknowledge the fundamental disconnect between the goals of industry (tobacco, alcohol, food) and of public health (Collin et al. 2017). Specifically to researchers, studies (including systematic reviews) indicate that—although industry-funded researchers acknowledge the potential for shifting agendas and risk to scientific integrity—they have less negative attitudes than those not funded by industry (Nestle 2016; Fabbri et al. 2018). Researchers who had more positive attitudes towards industry-funded research were more likely to accept industry funding (Fabbri et al. 2018).

Although research to date on attitudes of researchers to for-profit funded research has included health and public health research and researchers, the specific attitudes of public health academics have not been explored (Nakkash et al. 2016). COI and biases around FPO funding of research are particularly relevant when public health programmes, whose mission is to promote population health, receive funds from corporations whose products have negative public health implications. Our study aimed to assess attitudes and behaviours of public health academics globally towards receiving funding from FPOs for research, practice, and education.

## Methods

### Survey development and pilot testing

A cross-sectional survey was developed to assess attitudes and practices of public health academics towards accepting funding from FPOs. The survey questions were based on a literature review of results of research with a similar objective (Glaser and Bero 2005; Lipton et al. 2004; Abbas 2007; Harman 2001). The survey consisted of three sections (online resource):

1. A sociodemographic section asked about respondent characteristics (Table 1).

**Table 1:** Sample of universities, invited academics, and completed surveys by global region, 2017–2018

Region	Number (and per cent) of universities with public health programme that included emails on their website—*n* (%)	Number of academics whose emails were available and were invited to complete the survey—*n*	Completed surveys—*n* (%)	Number of institutions with which the respondents are affiliated—*n*	Per cent of completed surveys coming from each region
USA	40 (33.6%)	5037	92 (1.83%)	36	55.4%
EMR	22 (18.5%)	493	14 (2.84%)	7	8.4%
SEA	19 (16%)	498	11 (2.21%)	6	6.7%
AFR	13 (10.9%)	289	14 (4.84%)	8	8.4%
EUR^1^	25 (21%)	1020	35 (3.43%)	17	21.1%
Total	119	7337	166 (2.26%)	74	(100%)

*USA* United States of America, *EMR* Eastern Mediterranean Region, *SEA* Southeast Asia Region, *AFR* African Region, *EUR* European Region

^1^Based on WHO definition of European region (not EU definition)
2. A section that included 20 scenarios of possible industry-supported funding for public health-related research, practice, and education based on the models of funding described in Cohen et al. (2009) and specific examples found in the literature of funding relationships between academic institutions and corporations. The scenarios (listed in Table 4) varied along five features (online resource).
3. A section that assessed respondent attitudes and practices towards industry-sponsored research funding.
  - Attitude towards accepting funding from for-profit corporations. Responses options were yes under any circumstances, yes under certain circumstances, and no. Only five respondents stated they would take funds under any circumstance; thus, we combined their responses to those who answered ‘yes, under certain circumstances’ to create a dichotomous variables (yes, no).
  - Factors influencing decision to accept funds from FPOs
  - Potential for bias in research funded by for-profit corporations.
  - Practices:
    (i) Education around ethical issues in research.
    (ii) Received funding from FPOs.

We invited a convenience sample of 50 public health academic colleagues from the selected regions to pilot-test the survey (10 from each of five regions); 18 complete responses were received: 5 from colleagues in the USA; 4 each from Africa, Europe, and Southeast Asia; and 1 from the Eastern Mediterranean Region. Minor revisions were made to the survey based on the pilot test results.

### Respondent sampling strategy

The sampling frame was universities in five world regions—Africa, Eastern Mediterranean, Europe, Southeast Asia, and the USA (for North America)—that offered a graduate degree in public health. These regions were selected based on the expertise and location of co-investigators on this project. In Africa, the Eastern Mediterranean, Europe, and the USA, the sampling frame consisted of universities that were members of regional public health associations or networks, such as the Eastern Mediterranean Regional Academic Institutions’ Network (EMRAIN) for Public Health, the Council on Education for Public Health (CEPH), the Association of Schools of Public Health in Africa (ASPHA), and the Association of Schools of Public Health in the European Region (ASPHER). For Southeast Asia, the sampling frame started from the list of Asia–Pacific Academic Consortium for Public Health (APACPH) and also included a list of non-APACPH member Southeast Asian universities offering MPH degree for more than 1 year and with websites available in English language. From this total sampling frame, in all regions we selected all universities whose public health programme had a list of faculty members with emails on their website. For the USA, we capped recruitment at 40 universities. This resulted in a total sample of 119 (Table 1). Once the university sample had been identified, we progressed to forming the sampling frame of academics within those programmes of public health. This resulted in a list of 7337 academic public health researchers who were invited to complete the survey (Table 1).

### Survey administration

The survey was uploaded into LimeSurvey (an online survey platform) (LimeSurvey, n.d.) managed at the American University of Beirut. We sent email invitations to all 7337 academic public health researchers between December 2017 and May 2018. The invitation included information about the aim of the survey, details about the survey content, and information related to confidentiality and other ethical issues. While invitations were sent by email via LimeSurvey, emails were assigned random tokens with no identifiers. Each respondent received a maximum of three reminders, 1 week apart. If they chose to participate, they were directed to the consent form, followed by the eligibility questions and the survey questions. Respondents were eligible if they had been employed at their university for at least 1 year and had sought or received funds for research, education, or professional practice (service) in the last 5 years.

### Analysis

We conducted descriptive analysis of the survey results. Bivariate analyses were conducted between outcomes of interest, such as attitude and behaviour, and selected sociodemographic factors. We followed this with a logistic regression analysis to identify factors associated with accepting funds. We ran frequencies for the responses to the scenarios overall and bivariate analyses by region and by attitude. We subsequently ran logistic regression analyses for the scenario with the highest and lowest percentage of respondents who would accept the funds under those parameters to identify factors influencing decisions to accept or reject funding. All analyses were conducted in SPSS 25.

### Ethical approval

Ethical review and approval for the study was obtained from the institutional review board offices in each of the American University of Beirut, Lebanon; James P. Grant School of Public Health (JPGSPH), BRAC University, Bangladesh; University of Cape Town Health Sciences Faculty, South Africa; and the London School of Hygiene and Tropical Medicine, UK.

## Results

### Sociodemographic characteristics of respondents

A total of 166 eligible and complete responses were received giving a response rate of 2.26%. Over half (55.4%) of the responses (*n* = 92) came from academics in the USA, and another 21% (*n* = 35) from academics in Europe. We combined responses from the three other world regions (combined = 23.5% of respondents) and considered this group the low- and middle-income country (LMIC) region. LMIC respondents were more likely to be associate professors (rather than professors), to self-identify more often as teacher rather than researcher, and to be less likely to have any required salary offset. Table 2 provides detailed results of the sociodemographic characteristics of the respondents.

**Table 2:** Sociodemographic characteristics of participants (total *N* = 166) by global region, 2017–2018

Characteristic/region	USA%	Europe%	EMR/SEA/AFR%	Total% (*n*)**
Sex: % female	56	60	53.8	56.4 (165)
Age group				
25–34 years	6.6	5.7	2.6	5.5
35–44 years	17.6	25.7	35.9	23.6
45–54 years	26.4	25.7	33.3	27.9
55 + years	49.5	42.9	28.2	43.0 (165)
Current academic rank*				
Professor or Prof Emeritus	48.9	45.7	23.1	42.2
Associate Professor	22.8	37.1	43.6	30.7
Assistant Professor	22.8	5.7	15.4	17.5
Other	5.4	11.4	17.9	9.6 (166)
*Highest degree: % Ph.D.*	96.7	94.3	87.2	94.0 (166)
Contract status				
Tenured	57.6	71.4	51.3	59.0
Long term (7+ years)	7.6	2.9	20.5	9.6
Short term (3–6 years)	15.2	17.1	17.9	16.3
Shorter term (1–2 years)	19.6	8.6	10.3	15.1 (166)
% of salary offset required*				
0%	34.4	67.7	68.6	48.7
1–25%	10.0	6.5	11.4	9.6
26–50%	13.3	9.7	17.1	13.5
> 50%	42.2	16.1	2.9	28.2 (156)
Primary identity*				
Researcher	86.7	81.8	64.1	80.1
Practitioner	7.8	0.0	5.1	5.6
Educator/teacher	5.6	18.2	30.8	14.2 (162)

Italicized: more than 25% of cells have expected values < 5

*USA* United States of America, *EMR* Eastern Mediterranean Region, *SEA* Southeast Asia Region, *AFR* African Region, *EUR* European Region

*p < 0.01 **Ns are totals for all options of the variable, e.g. sex *n* = for males and females

### Attitudes towards accepting funding from for-profit corporations

Over half of respondents (54%) stated they were in favour of accepting funds from FPOs for research and practice. We found significant differences in attitude by region: 31% (*n* = 11) of respondents from the European region stated that they were in favour of accepting funds (vs. 62% from the USA and 54% from the LMICs). Females (45%, *n* = 42) were significantly less likely to favour accepting such funds (vs. 64% of males, *n* = 46). Respondents with higher required salary offset were significantly more likely to favour accepting such funds than those with less required salary offset. We found no statistically significant differences in attitudes by academic rank, contract status, and primary identity (Table 3). Further, no significant differences in attitudes were found if respondents had received training on research ethics (versus not), taken an online ethics course (versus not), or attended a conference on the ethics of receiving funds from for-profit companies (versus not) (Table 3).

**Table 3:** Attitude and behaviour regarding accepting funds from for-profit organizations by selecting sociodemographic characteristics, 2017–2018

	In favour of accepting funds from for-profit organizations? %		Funded by a for-profit organization for research or practice? %	
	Yes	No	Yes	No
Region				
USA	62.0*	38.0	25.0	75.0
Europe	31.4	68.6	14.3	85.7
EMR/SEA/AFR	53.8	46.2	12.8	87.2
Sex				
Male	63.9 Ω	36.1	30.6*	69.4
Female	45.2	54.8	11.8	88.2
Current academic rank				
Professor/Prof Emeritus	51.4	48.6	21.4	78.6
Associate Professor	51.0	49.0	13.7	86.3
Assistant Professor	58.6	41.4	24.1	75.9
Other	37.5	62.5	25.0	75.0
Contract status				
Tenured	46.9	53.1	18.4	81.6
Long term (7 + years)	56.2	43.8	25.0	75.0
Short term (3–6 years)	59.3	40.7	18.5	81.5
Shorter term (1–2 years)	72.0	28.0	24.0	76.0
% of salary offset required				
0%	46.1 Ω	53.9	13.2	86.8
1–25%	33.3	66.7	20.0	80.0
26–50%	57.1	42.9	33.3	66.7
> 50%	72.7	27.3	27.3	72.7
*Primary identity*				
Researcher	*52.3*	*47.7*	17.7	82.3
Practitioner	*77.8*	*22.2*	22.2	77.8
Educator/teacher	*52.2*	*47.8*	30.4	69.6
Received training on research ethics				
Yes	53.9	46.1	20.8	8.3
No	50.0	50.0	79.2	91.7
Completed research ethics online course				
Yes	56.5	43.5	22.6	11.9
No	45.2	54.8	77.4	88.1
Attended conference on ethics of receiving for-profit funding				
Yes	58.5	41.5	75.6	81.6
No	52.0	48.0	24.4	18.4

Italicized: more than 25% of cells have expected values < 5

*USA* United States of America, *EMR* Eastern Mediterranean Region, *SEA* Southeast Asia Region, *AFR* African Region, *EUR* European Region

*p ≤ 0.01/Ω *p* ≤ 0.05

The logistic regression confirmed the results of the bivariate analysis. When compared to respondents in the USA, those in Europe were less likely to favour accepting funds from FPOs for research and practice (OR = 0.23; *p* = 0.016). In addition, compared to males, females were less likely to favour accepting such funds (OR = 0.33; *p* = 0.006) (Table 4).

**Table 4:** Logistic regression for variables predicting an attitude favouring accepting funds from for-profit organizations, 2017–2018

Variable	OR (95% CI)	*p* value
Region (base: USA)		
Europe	0.23 (0.07–0.76)	0.016
EMR/SEA/AFR	0.51 (0.17–1.54)	0.232
Gender (base: male)		
Female	0.33 (0.15–0.72)	0.006
Current academic rank (base: prof/prof emeritus)		
Associate professor	1.90 (0.75–4.77)	0.174
Assistant professor	2.35 (0.81–6.81)	0.117
Other	4.20 (0.99–17.69)	0.051
% of salary offset required (base: 0%)		
1–25%	0.40 (0.11–1.51)	0.178
26–50%	1.17 (0.38–3.63)	0.781
Over 50%	2.24 (0.88–5.72)	0.091
Primary identity (base: researcher)		
Practitioner	2.43 (0.42–14.08)	0.323
Teacher/lecturer/instructor	1.07 (0.35–3.25)	0.905
Received training on research ethics (base: no)		
Yes	1.61 (0.35–7.37)	0.539
Completed research ethics online course (base: no)		
Yes	0.61 (0.20–1.89)	0.390
Attended conference on ethics of receiving for-profit funding (base: no)		
Yes	1.24 (0.51–3.03)	0.631

*USA* United States of America, *EMR* Eastern Mediterranean Region, *SEA* Southeast Asia Region, *AFR* African Region, *EUR* European Region

### Factors influencing decision about accepting funds from for-profit corporations

About three-quarters (72%; *n* = 120) of respondents stated that the ‘type of product’ would influence their decision-making. We followed up on types of product: 74% of respondents stated they would accept funds from health insurance companies, 65% from pharmaceutical companies, 44% from the petroleum industry, 36% from food and sweetened beverage companies, 25% from gambling companies, 25% from the alcohol industry, 12.5% from the tobacco industry, and 10% from arms dealers and manufacturers. In addition, 67% (*n* = 112) of respondents stated that the intended use of the funds would influence their decision-making: of these, 85% (*n* = 95) stated they would accept funds for research, 82% (*n* = 92) for scholarships, 68% (*n* = 76) for facilities including equipment, 35% (*n* = 39) for sponsorship, and 27% (*n* = 30) for salaries. Seventeen per cent of respondents (*n* = 29) stated that the size (amount) of the grant would influence their decision-making. However, most of these respondents (*n* = 24) did not identify any threshold amount which would influence their decision to accept funding. Other influential factors were the terms and conditions of the funder (for 50% of the 120 respondents), their university funding policy (67%), if the grant supports the institution’s mission (50%), their career trajectory/promotion cycle (7%), and the stability of their income (4%) [data not shown].

Respondents were asked whether they felt that—when funded by FPOs, there was potential for bias (to a great extent or some extent) resulting from seven aspects of research: research design; analysis of data; outcomes/results of the data; delays in publications; control over access to tools; limiting access to data; and engaging underqualified or easily influenced researchers. Seventy-three percent agreed that there was potential for bias in 6 or 7 of these aspects of research. Over half (52.4%) of these respondents stated that they were not in favour of accepting funds for research or practice from FPOs as opposed to 29.7% of respondents who agreed there was potential for bias in less than 6 aspects of research (*p* < 0.05) [data not shown].

Overall, 94% of respondents agreed that it would be useful to have university guidelines that govern accepting funds from FPOs, and 52% of respondents felt that the university should set these guidelines, while 24% thought an international public health association should do so.

### Receipt of funds from for-profit corporations in the last 5 years

In the last 5 years, almost 20% (33/166) of the respondents had received funding from FPOs. We found differences by sex with males more likely to have received such funding. We found no significant differences by any other variable. Indeed, the data on exposure to ethics content tended to trend in the opposite direction than expected (though not significant) with those respondents who had been exposed to ethical educational content being more likely to have been funded by FPOs (Table 3).

When asked about the kind of FPO that funds had been received from, none stated tobacco, alcohol, or gambling; 8.4% (*n* = 14) received funds from pharmaceuticals, 4.2% (*n* = 7) from food and sweetened beverages organizations, 3.6% (*n* = 6) from health insurance companies, 1.8% (*n* = 3) from petroleum companies, and 0.6% (*n* = 1) from arms dealers and manufacturers [data not shown].

### Responses to scenarios

Overall, there were seven scenarios that would result in over 50% of respondents refusing funding (Table 5). These scenarios spanned a variety of types of products, as well as grant amount, nature of funded activities, and target population. However, respondents seemed most likely to state that they would refuse funding when the topic was directly related to the core business of the industry (e.g. a tobacco company sponsoring tobacco control research; scenarios 2, 5, 6, 7), or when the scenario specifically suggested a negative human rights or environmental practice by the industry (e.g. mistreating workers; scenarios 5, 14, 19). Scenarios where at least 80% of respondents did not reject funding (i.e. would accept or were not sure) were more likely to be scenarios where organizations were (a) at arms length from the funded activity (scenario 8) or (b) involved in activities not directly related to the funded activity (scenario 16), or (c) seemed to be advancing a commitment to health in the organization itself (scenario 18).

**Table 5:** Percentage of respondents who would definitely refuse or refuse to accept funds based on the following scenario overall and by global region and attitude, 2017–2018

Scenario	Overall refuse (%)	Refuse (%) by region			Refuse (%) by attitude: In favour of accepting funds from for-profit organizations?	
		USA	Europe	EMR/SEA/AFR	Yes	No
6. A tobacco company offers you funding for a study investigating the impact of tobacco products and e-cigarettes	77.4	77.5	81.8	73.0	68.6*	87.8
9. A billionaire, whose wealth comes primarily from arms sales, wants to donate money to construct a building in your university with his name on it	71.2	65.1	87.9	70.3	59.5 Ω	84.7
14. A warehouse department store, whose employees suffer from exploitation and violence at the workplace, wants to donate $10’000 for facilities and equipment to your faculty	68.1	63.6	79.4	68.4	56.8 Ω	81.9
5. A corporation in the sports clothing industry with factories in third world countries with a questionable environmental record wants to sponsor a ‘greening the environment’ initiative at your university	61.9	59.3	75.0	56.8	48.8 Ω	76.7
7. A multinational corporation that manufactures soft drinks, juices, and packaged junk food is seeking to recruit Public Health researchers from your faculty in order to conduct a study on fitness and other health-related topics for children	56.3	55.7	57.6	56.8	35.3 Ω	80.8
19. A multinational phone company wants to donate $500’000 to support a project assessing risks related to child labour that your faculty is conducting. This company has recently been in the news for exploiting their workers	53.8	46.0	64.7	62.2	45.9 Ω	63.0
2. A soft drink beverage company wants to fund an intervention in your faculty aimed at promoting healthy eating	52.8	46.7	67.6	53.8	34.1 Ω	74.7
11. A pharmaceutical firm recently fought against its drugs being manufactured in India as generics, arguing patent and intellectual property rights. It wants to support research in health policy at your university	49.1	44.4	61.8	48.6	36.8 Ω	63.5
15. A fast-food corporation wants to donate $5’000 for a one-day students’ health education activity organized by your Public Health School	45.6	37.9	58.8	51.3	32.6 Ω	60.8
12. An alcohol industry donates money to your university’s Office of Grants. The office will be responsible for the distribution and allocation of the money for various projects without directly acknowledging the alcohol industry’s involvement	44.7	33.0*	**60.6**	**57.9**	33.7 Ω	57.5
13. An international tobacco company, in partnership with the International Labour Organization, wants to fund an advocacy campaign in order to stop the exploitation of child labourers in tobacco farming and approaches you to plan and evaluate such a campaign	44.7	36.4	58.8	51.4	31.0 Ω	61.1
3. A pharmaceutical company that recently developed nutritional supplements wants to fund an intervention at your faculty aimed at promoting exercise	37.7	38.9	29.4	42.1	23 Ω	54.7
17. A company that manufactures fertilizers and pesticides wants to sponsor a research study your faculty is conducting on farmers’ protective clothing	36.3	35.6	39.4	35.1	22.4 Ω	52.8
20. A gambling company wants to donate $1.2 million to your university’s art and music department. The donation will go towards an initiative the arts/music department is working on to build a visual and performing arts centre for the youth in an impoverished neighbourhood of the university	34.2	21.6*	**55.9**	**44.4**	24.4 Ω	45.8
4. A billionaire, whose wealth comes primarily from telecommunications but who also has investments in tobacco companies, wants to set up a family health centre at your university to support innovative programmes in maternal and child health	32.1	20.0*	**50.0**	**44.7**	17 Ω	50.0
1. A fast food corporation wants to provide an anonymous full scholarship to financially disadvantaged, yet academically promising, students for a degree in public health at your university	28.6	18.2*	**44.1**	**38.5**	14.0 Ω	45.3
10. A pharmaceutical company that manufactures chemotherapy drugs wishes to sponsor an intervention campaign to screen for breast cancer at your university’s infirmary	25.5	28.1	21.9	21.1	16.5 Ω	36.1
18. A recognized foundation recently divested from its tobacco stocks wants to fund a smoking cessation programme that is being implemented at your university	18.5	9.2*	**29.4**	**30.6**	11.5 Ω	27.1
8. A financial services corporation establishes a foundation with its namesake but with an independent Board of Trustees. This foundation wants to sponsor fellowships in health care financing at your university	14.4	8.9	25.0	18.4	9.1 Ω	20.8
16. An international businessman who manages global investments in oil and gas wants to donate $20 million to the renovation and expansion of your university medical centre	14.2	**7.0 Ω**	**27.3**	**19.4**	8.2 Ω	21.4

Bolded percentages indicate significant differences between regions

*USA* United States of America, *EMR* Eastern Mediterranean Region, *SEA* Southeast Asia Region, *AFR* African Region, *EUR* European Region

*p ≤ 0.01/Ω *p* ≤ 0.05

We explored differences by region. In 17 of the 20 scenarios, respondents from Europe were more likely than those from the USA and the LMICs to refuse funding; and in 15 of the scenarios, respondents from the USA were least likely to refuse the funding. There were significant differences between regions to six of the scenarios (bolded in Table 5). In all six, respondents from the USA were significantly less likely to refuse the funding.

We also explored differences by attitude towards accepting funds from FPOs. For all the scenarios, those who were not in favour of accepting such funds were significantly more likely to state that they would refuse receiving funding.

We ran logistic regressions including all the sociodemographic variables for the scenario with the highest (Scenario 6) and lowest (Scenario 16) percent of respondents stating that they would refuse funding (Table 6). There were no variables that significantly differentiated responses for Scenario 6. Only region was significantly difference for Scenario 16. Compared to respondents in the USA, those in Europe were more likely to refuse the funding (OR = 5.58; *p* = 0.036).

**Table 6:** Logistic regression for variables predicting refusal to accept funds for scenario with the highest percent refusal overall and lowest percent refusal overall, 2017–2018

Variable	Scenario 6 (see Table 5)		Scenario 16 (see Table 5)	
	OR (95% CI)	*p* value	OR (95% CI)	*p* value
Region (base: USA)				
Europe	3.44 (0.73–16.09)	0.117	5.58 (1.11–27.94)	0.036
LMICs	1.10 (0.30–4.03)	0.888	3.98 (0.79–19.90)	0.093
Gender (base: male)				
Female	1.08 (0.44–2.62)	0.866	1.65 (0.54–5.02)	0.376
Current academic rank (base: prof/prof emeritus)				
Associate professor	0.91 (0.31–2.67)	0.859	0.69 (0.18–2.62)	0.581
Assistant professor	1.87 (0.50–6.95)	0.348	1.09 (0.22–5.30)	0.918
Other	0.71 (0.15–3.30)	0.662	0.36 (0.03–3.97)	0.402
% of salary offset required (base: 0%)				
1–25%	0.64 (0.16–2.56)	0.531	0.52 (0.06–4.77)	0.560
26–50%	0.84 (0.22–3.22)	0.801	0.96 (0.16–5.94)	0.965
Over 50%	0.90 (0.30–2.68)	0.845	1.18 (0.27–5.14)	0.822
Primary identity (base: researcher)				
Practitioner	2.79 (0.31–25.48)	0.362	1.64 (0.16–16.81)	0.676
Teacher/lecturer/instructor	0.41 (0.12–1.36)	0.145	1.20 (0.28–5.15)	0.803
Received training on research ethics (base: no)				
Yes	1.89 (0.30–11.93)	0.497	0.72 (0.10–5.43)	0.752
Completed research ethics online course (base: no)				
Yes	0.89 (0.22–3.52)	0.864	1.19 (0.29–4.89)	0.806
Attended conference on ethics of receiving for-profit funding (base: no)				
Yes	0.64 (0.24–1.73)	0.378	0.34 (0.06–1.74)	0.193

## Discussion

Our study assessed the attitudes and behaviours of public health academics globally towards accepting funding for research and practice from FPOs. Research on this topic specifically focused on public health academics is still nascent (Nakkash et al. 2016). Overall, over half of our respondents were in favour of accepting this funding. This is concerning given that many of these FPOs market products that have negative health consequences; and given evidence that research funded by FPOs is biased in favour of the corporation’s products (Nestle 2016; Bero et al. 2007; Lundh et al. 2017; Moynihan et al. 2019; Babor and Robaina 2013). Research in the University of California system has found similar attitudes among academics generally (not specifically public health) (Lipton et al. 2004). However, a survey of health researchers, advocates, and policymakers in 40 countries found overwhelming agreement that there was a ‘conflict between industry objectives and public health objectives’ (Collin et al. 2017).

Our results further suggest that only region, gender, and % salary offset were significantly related to attitude towards accepting funds. In contrast, when asked whether industry sponsorship of research was necessary, respondents from medical institutions in both developing and developed countries overwhelmingly agreed (84%), with no differences between them (Abbas 2007).

Our respondents generally had more favourable attitudes towards accepting funding from certain FPOs over others, depending on their ‘product’, with alcohol, gambling, tobacco, and arms receiving the least favourable responses. Collin et al. (2017) similarly found stronger negative reactions towards partnership with alcohol and tobacco companies than food companies. Notwithstanding, these findings are of great concern given documented actions by all these FPOs to negatively impact health outcomes and to interfere with research. One of the key strategies employed by FPOs to influence policy and protect their interests is to influence research and the production of evidence (Fabbri et al. 2018), including challenging independently produced evidence, aiming to discredit the quality of the research, and the reputation of the researcher(s); and producing and disseminating irreproducible research (Savell et al. 2014, 2016; Petticrew et al. 2012).

Specific to certain ‘products’, responses of participants in our research to the three scenarios linked to pharma indicated refusal rates for funding from pharmaceuticals of at most 50% (scenarios 3, 10, 11). This, despite the fact that the pharmaceutical industry, currently facing multiple lawsuits due to their complicity in the opioid epidemic, has a history of interference in health research and policy (Sismondo 2008; Bero et al. 2007; Dyer 2020). The alcohol industry has successfully infiltrated government research bodies and established partnerships to bolster its influence (Readon 2018; Paixão and Mialon 2019); yet, less than half of our respondents stated that they would refuse funding from the alcohol industry (scenario 12). With regard to tobacco, although our respondents were most likely to refuse one of the tobacco-related scenarios (scenario 6), other such scenarios seemed to be seen as more acceptable (scenarios 13, 18). These scenarios differed on several of the features, e.g. nature of the funded activity, type of grant provider. The tobacco industry has stymied tobacco control efforts globally (Malone et al. 2017).

Despite these attitudes that seem somewhat accepting of funding from FPO, the majority of our respondents acknowledged that such funding leads to bias in multiple aspects of research. Our results also corroborate other studies of researcher perceptions of bias (Lipton et al. 2004). As a result of these perceptions of bias, almost all our respondents agreed that it would be useful to have university guidelines to govern receipt of these funds. Several such guidelines have been proposed (Cohen et al. 2009; Adams 2007). More recently, a recommendation has been made for the elimination of any research funding relationships with FPOs (Moynihan et al. 2019; Goldberg 2019).

Interestingly, exposure to research ethics education had no bearing at all on any of the outcomes described above (attitude, behaviour, and scenario response). Although our non-significant results could be related to the temporality of the questions around ethics (ever), and the response to the attitude and scenario questions (the present), as well as the behaviour (past 5 years), it remains concerning that exposure was unrelated to attitude or behaviour. This suggests that research ethics training may not necessarily create a more reflective researcher or practitioner. Evaluation research on the impact of ethics training suggested that, while knowledge gain was the most salient outcome, no or limited improvement in moral reasoning was demonstrated (Rosenbaum 2003; Schmaling and Blume 2009). One possible explanation for limited impact on attitudes and behaviours could be that ethics instruction may make people feel immune to risky behaviour (Antes et al. 2010). In fact, ethics training in some instances had unintended consequences and was harmful as students expressed inflated confidence in their problem-solving skills when it came to ethical issues (Kalichman 2013). Although none of these studies specifically tackled ethics of FPO funding, their results on the difficulty of changing attitude and behaviour are likely generalizable. It may be timely to review online and curricular research ethics course content and instructional method—to ensure adequate coverage of topics around governance, ethics, and COI in the interaction between public health research, practice, and policy and for-profit corporations, and enhance their capacity to influence attitudes and behaviours.

Finally, in our sample of surveyed public health professionals, almost 20% reported receiving funding from FPOs—usually pharmaceutical companies—in the past years, with males being more likely to have done so. Previous studies have reported a wider range reporting current funding from industry—17%–70%, depending on region and type of professional (Abbas 2007; Harman 2001; Blumenthal et al. 1986a, b). This topic is perceived to be sensitive and inflammatory which also may explain the wide range of responses across studies. Some of our respondents indicated that the results of their FPO funded research had been unfavourable to the funding organization, but most stated that the results were still published without delay. Rasmussen et al. (2018) found that only 33% of academic researchers indicated that they had the final say in the design of studies funded by FPOs, but did not report delays in publication or disagreements with funders.

Our study failed to obtain the representative sample intended from the five regions. In the Americas, we only focused on North America. Many universities in LMICs did not have websites, or when they did, did not include email addresses of their faculty members on the website, making the creation of a comprehensive sampling frame difficult. Also, for researchers that did receive the survey, very few—particularly from the global South—completed it, potentially due to the sensitive nature of these questions. We recognize that this limits the generalizability of the findings, but we believe our findings fill an important gap in the literature given the dearth of research in this topic area. A non-random sampling strategy was also necessary in other similar research (Collin et al. 2017). Moreover, despite this limitation, at least one person replied from 74 out of the 119 institutions that were included in our sampling frame. Future research should consider actively engaging associations of schools of public health in all regions where these exist, rather than only contacting the member institutions, as we did. This may provide ease of access, increased legitimacy, and response rates, but may bring its own biases. In addition, we used an online survey, which has been suggested as an alternative method to traditional surveying, though not without its own biases (Braithwaite et al. 2003).

These results of this study suggest the need for increasing dialogue in public health academia around the potential harms of research and practice funded by FPOs whose products have negative public health consequences. Recently, a global network entitled ‘Governance, Ethics, and COI in the interaction of industry and public health research, practice and policy (GECI-PH)’ has been established. It consists of over 80 academics, researchers, and practitioners committed to controlling FPO influence on public health. Our study further suggests the imperative for universities to develop policies on whether and how to accept such funds. Potential avenues for further research can include (1) conducting systematic reviews of the literature on the methods and/or effects of FPO influence on research and the production of evidence; (2) research on the usability of the research produced, and how users of such evidence—such as policymakers and practitioners—should assess its validity and susceptibility to bias—other than by using standard critical appraisal tools; (3) qualitative research with academic and non-academic (e.g. research councils) stakeholders, and in different geographical regions to gain their views and experience on this subject, and to further inform the areas for analyses; (4) evaluation of ethics training for its impact on attitudes and behaviours related to accepting funding from FPOs. Finally, we call on public health academic associations to develop specific public health degree education competencies to ensure awareness of the potential biases and concerns related to for-profit corporation interference in public health education, research, practice and policy; and endorsement of attitudes refusing such engagement.

## Electronic supplementary material

Below is the link to the electronic supplementary material.

Supplementary material 1 (PDF 123 kb)
