# Supplementary material for: Attitudes and practices of public health academics towards research funding from for-profit organizations: cross-sectional survey
Source: Int J Public Health. 2020 Aug 25;65(7):1133–45. doi: 10.1007/s00038-020-01416-0 (PMC7497330; doi:10.1007/s00038-020-01416-0)
Supplement: Supplementary file 1 — Supplementary material 1 (PDF 123 kb) [file 38_2020_1416_MOESM1_ESM.pdf]

International Journal of Public health

Article title: Attitudes and practices of public health academics towards research funding from for-profit organizations: Cross-sectional survey

Table A: Online Survey (English version), 2017-2018

PART I: DEMOGRAPHIC INFORMATION

This section asks general information about you. Please select the answer that most describes you.

1. What is the name of the public health institution where you currently work? (please write the full name)
    a. open ended

2. In which region is your institution located?
    a. Drop down

3. In what country is your institution located?
    a. Drop down

4. What is your gender?
    a. Male
    b. Female
    c. Other

5. To which age group do you belong?
    a. 25-34 years
    b. 35-44 years
    c. 45–54 years
    d. 55–59 years
    e. 60 years or more

6. What is your current Academic Rank?
    a. Professor emeritus
    b. Professor
    c. Associate Professor
    d. Assistant Professor
    e. Lecturer
    f. Instructor
    g. Research associate /fellow
    h. Academic Program coordinator
    i. Academic Project coordinator
    j. Academic Project associate
    k. Academic Project assistant
    l. Other (specify) ________________________________

7. What are the highest degrees you attained? (checkall that apply)
    a. Doctorate (PhD, DrPh, MD, PharmDm etc)
    b. MPH
    c. MS/MA in public health
    d. Other MS/MA
    e. BS/BA in Public Health
    f. Other BS/BA
    g. Other (specify) ________________________________

8. How many years have you been employed at your current institution?
    a. 1–2 years
    b. 3-6 years
    c. 7 -12 years
    d. 13 years or more

9. How much of your salary do you have to raise yourself to remain employed by your institution?
    a. 0%
    b. 1-25%
    c. 26-50%
    d. Over 50%

10. What is your contract status?
    a. Tenured
    b. Long term contract (7 years or more)
    c. Short term contract (3-6 years)
    d. 1-2 year contract

11. Based on the definitions below, do you identify as a researcher, teacher or practitioner? (check all that apply)

    a. Researcher
    b. Practitioner
    c. Teacher/ lecturer/ instructor

**A researcher**: Pursues careers in research, or university teaching in graduate programs, policy analysis and development.<br>**A teacher:** Pursues a career in teaching.<br>**A practitioner**: Pursues careers in the practice of public health to enhance the wellbeing of the individual, community, groups, and populations through implementing community based intervention using epidemiological investigation, surveillance…

PART II: Scenarios

This section consists of 20 short scenarios that aim to capture several variables that affect people's decisions regarding accepting funds for research and practice. These variables include:

- ❖ Size of grant in USD (< 50,000; 50-99 K; 100-249 K; 250-499 K; 500-999 K; 1 million or more)
- ❖ Type of grant (individual; research team; research center; endowed chair)

- Type of for-profit organization (food and beverage; pharmaceutical; tobacco industry; petroleum; health insurance, gambling, alcohol industry, arm dealing and manufacturing)
- Target population (developing countries, disadvantaged communities; women; children.)
- Type of research (basic science; clinical; population-based; policy; intervention)

For each scenario proposed, please choose a number from 1 to 5 that illustrates your decision regarding taking funds from for profit organizations given the specific variables described in each scenario.

5 = Definitely Accept
4 = Accept
3 = Not Sure
2 = Refuse
1= Definitely Refuse

SCENARIOS		Definitely refuse	Refuse	Not sure if I would accept or refuse	Accept	Definitely accept	Briefly explain your choice
		(1)	(2)	(3)	(4)	(5)	
1.	A fast-food corporation wants to provide an anonymous full scholarship to financially disadvantaged, yet academically promising, students for a degree in public health at your university.						
2.	A soft-drink beverage company wants to fund an intervention in your faculty aimed at promoting healthy eating.						
3.	A pharmaceutical company that recently developed nutritional supplements wants to fund an intervention at your faculty aimed at promoting exercise.						
4.	A billionaire, whose wealth comes primarily from telecommunications but who also has investments in tobacco companies, wants to set up a family health centre at your university to support innovative programs in maternal and child health.						
5.	A corporation in the sports clothing industry with factories in third world						

SCENARIOS		Definitely refuse	Refuse	Not sure if I would accept or refuse	Accept	Definitely accept	Briefly explain your choice
		(1)	(2)	(3)	(4)	(5)	
	countries with a questionable environmental record wants to sponsor a ‘greening the environment’ initiative at your university.						
6.	A tobacco company offers you funding for a study investigating the impact of tobacco products and e-cigarettes.						
7.	A multinational corporation that manufactures soft drinks, juices and packaged junk food is seeking to recruit Public Health researchers from your faculty in order to conduct a study on fitness and other health related topics for children.						
8.	A financial services corporation establishes a foundation with its namesake but with an independent Board of Trustees. This foundation wants to sponsor fellowships in health care financing at your university.						
9.	A billionaire, whose wealth comes primarily from arms sales, wants to donate money to construct a building in your university with his name on it.						
10.	A pharmaceutical company that manufactures chemotherapy drugs wishes to sponsor an intervention campaign to screen for breast cancer at your university’s infirmary.						
11.	A pharmaceutical firm recently fought against its drugs being manufactured in India as generics, arguing patent and intellectual property rights. It wants to						

	SCENARIOS	Definitely refuse	Refuse	Not sure if I would accept or refuse	Accept	Definitely accept	Briefly explain your choice
		(1)	(2)	(3)	(4)	(5)	
	support research in health policy at your university.						
12.	An alcohol industry donates money to your university's Office of Grants. The office will be responsible for the distribution and allocation of the money for various projects without directly acknowledging the alcohol industry's involvement.						
13.	An international tobacco company, in partnership with the International Labour Organization, wants to fund an advocacy campaign in order to stop the exploitation of child labourers in tobacco farming and approach you to plan and evaluate such a campaign.						
14.	A warehouse department store, whose employees suffer from exploitation and violence at the workplace, wants to donate $10'000 for facilities and equipment to your faculty.						
15.	A fast-food corporation wants to donate $5'000 for a one-day students' health education activity organized by your Public Health School.						
16.	An international businessman who manages global investments in oil and gas wants to donate $20 million to the renovation and expansion of your university medical Center.						
17.	A company that manufactures fertilizers and pesticides wants to sponsor a						

	SCENARIOS	Definitely refuse	Refuse	Not sure if I would accept or refuse	Accept	Definitely accept	Briefly explain your choice
		(1)	(2)	(3)	(4)	(5)	
	research study your faculty is conducting on farmers' protective clothing.						
18.	A recognized foundation recently divested from its tobacco stocks, wants to fund a smoking cessation program that is being implemented at your university.						
19.	A multinational phone company wants to donate $500'000 to support a project assessing risks related to child labor that your faculty is conducting. This company has recently been in the news for exploiting their workers.						
20.	A gambling company wants to donate $1.2 million to your university's art and music department. The donation will go toward an initiative the arts/music department is working on to build a visual and performing arts centre for the youth in an impoverished neighbourhood of the university.						

PART III: ATTITUDES

This section asks about your general attitudes regarding the receipt of funds from for profit organization. We use this term to mean any national or international organization that sells consumer products related to food and beverages, tobacco, alcohol, and other organizations like pharmaceutical, gambling, arms dealing or manufacturing, health insurance companies and the petroleum industry.

1. Generally, are you in favor of accepting funds from for-profit organizations for research/practice?
    a. Yes
        i. Under any circumstances
        ii. Under certain circumstances (explain in the narrative box)
    b. No

2. What are the factors that influence your decision about accepting funds from for-profit organizations? (check all that apply)

a. Type of product (tobacco, sweetened beverage, pharmaceutical…)
b. The intended use of money (for research, facilities, sponsorship, scholarship…)
c. The size (amount) of the grant
d. Terms and conditions of the funder
e. The university funding policy
f. The Promotion cycle/ career trajectory
g. If the grant supports the Faculty/institution's mission
h. The stability of my income

3. If you chose answer 2a, which for-profit companies would you accept funds from? (check all that apply)
    a. Tobacco Industries
    b. Gambling Industries
    c. Alcohol Industries
    d. Pharmaceutical Industries
    e. Food and Sweetened Beverage Industries
    f. Arms Dealing and Manufacturing
    g. Health insurance companies
    h. Petroleum industry
    i. Other (specify) ____________________

4. If you chose answer 2b, for what purpose would you accept funds from for-profit organizations?
    a. Research
    b. Facilities (including equipment)
    c. Sponsorship
    d. Scholarship
    e. Salaries
    f. Other (specify) ____________________

5. If you chose answer 2c, Is there a threshold below which you do not apply/accept for funds?
    a. Yes
        i. Please specify the amount:
    b. No

6. If you choose answer 2f, more likely to accept if I am
    a. An assistant professor seeking funds to support my research or practice portfolio for promotion to associate
    b. An associate professor seeking funds to support my research or practice portfolio for promotion to a full professor
    c. A full professor

7. How likely is it that there is potential **bias** in the following aspects of research associated with taking funds from for-profit organizations?	To a great extent	To some extent	Not at all
**I believe that there is potential for bias:**			
In research design			

In the analysis of data			
In outcome/results of the data			
Leading to publication delay/suppression			
Leading to organization control over access to Research tool/material			
In case the organization limits access to data			
In case the organization will engage underqualified or easily influenced researchers			

8. Do you think it would be useful to have guidelines at universities that govern accepting funds from for profit organizations?
    a. Yes
    b. No

9. Who should set those guidelines?
    c. The university
    d. An international public health association
    e. Researchers/ practitioners/ teachers
    f. industry
    g. government
    h. Other

10. Would you take money from for-profit organizations as part of their corporate social responsibility (CSR)?
    a. Yes
        i. Under any circumstances
        ii. Under certain circumstances (narrative box)
    b. No

PART IV: Research and professional practice activities

This section asks about your research and professional practice activities. Please check the answer that best describes your activities (or more than one answer when so stated).

1. How often do you apply for funds for **research**?
    a. Once per year
    b. At least twice per year
    c. On an as needed basis

2. How often do you apply for funds for your professional practice work in public health (**other than research**)?
    a. Once per year
    b. Twice per year
    c. On an as needed basis

3. How many **scholarly and/or scientific articles** have you published during your career?
    a. Fewer than 5
    b. 6 to 10
    c. 11 to 20

d. 21 to 30
e. More than 30
f. Not applicable

4. How many publications – **other than research** – such as policy brief, newspapers articles, and/or reports have you published during your career?
    a. Fewer than 5
    b. 6 to 10
    c. 11 to 20
    d. 21 to 30
    e. More than 30
    f. Not applicable

5. How many of your **scholarly and/or scientific articles** are related to projects that were directly or indirectly funded by for-profit organizations?

    ____

6. How many of your publications, **other than research** (policy brief, newspapers articles, reports) are related to projects that were funded by for-profit organizations?

    ____

7. Have you ever received training on research ethics?
    a. Yes
    b. No

8. Have you ever had to complete a research ethics online test like (CITI or NIH)?
    a. Yes
    b. No

9. Have you ever attended a conference or meeting on ethics about receiving funds from for profit organizations?
    a. Yes
    b. No

10. Have you been funded to do public health research or practice by a for-profit organization during the past 5 years (including any now)?
    d. Yes
    e. No (Skip Q 11-15)

11. What kind of for profit organizations funded your projects? (check all that apply)
    a. Tobacco Industries
    b. Gambling Industries
    c. Alcohol Industries
    d. Pharmaceutical Industries
    e. Food and Sweetened Beverage Industries
    f. Arms Dealing and Manufacturing
    g. Health insurance companies
    h. Petroleum industry

i. Other (specify) ______________________

12. What was the process for obtaining the funds for your latest research/practice project funded by for-profit organizations?
    a. The organization approached me first
    b. I approached them first
    c. Other (specify) ______________________

13. Who wrote the proposal?
    a. I wrote the proposal
    b. The organization wrote the proposal
    c. We both wrote the proposal
    d. Other (specify) ______________________

14. Did this project need clearance from the University?
    a. Yes
    b. No (skip Q15)
    c. Not sure (skip Q15)

15. Who is the party at the university that granted you clearance?
    a. Institutional Review Board
    b. Research committee
    c. Other (specify) ______________________

16. Were any of the results in your organization-funded research unfavorable to the for-profit organization?
    a. Yes
    b. No (skip 17-18)

17. Were the results published
    a. Yes
    b. No

18. Was there a delay in their publication?
    a. Yes
    b. No

19. Did you feel at any time that there was any type of bias associated with the research / practice project? Bias could include (research design, analysis of data , outcome/results of the data , Publication delay/suppression for organization control over access to Research tool/material, for the organization limiting access to data, that organizations will engage under-qualified or easily influenced researchers to do their research)
    a. Yes
    b. No

20. If yes, can you explain briefly what type of bias
    ____
    ____
    ____

____

____

21. Would you take funds from for profit organizations for public health research or practice in the future?
    a. Yes
        i. Under any circumstances
        ii. Under certain circumstances (explain the circumstances)
    b. No

Thank you for completing this survey.

International Journal of Public health

Article title: Attitudes and practices of public health academics towards research funding from for-profit organizations: Cross-sectional survey

Table B: Matrix of scenario characteristics, 2017-2018

	SCENARIOS	Size of grant in USD (< 50,000; 50-99 K; 100-249 K; 250-499 K; 500-999 K; 1 million or more)	Type of grant (individual; research team; research center; endowed chair)	Type of for-profit organization (food and beverage; pharmaceutical; tobacco industry; petroleum; health insurance, gambling, alcohol industry)	Target population (disadvantaged communities; women; children; specific diseases or health outcomes; developing countries, arm dealing and manufacturing,)	Type of research/practice (basic science; bedside (patients/hospitals); population-based; policy; intervention)
21.	A fast-food corporation wants to provide an anonymous full scholarship to financially disadvantaged, yet academically promising, students for a degree in public health at your university.			x	x	x
22.	A soft-drink beverage company wants to fund an intervention in your faculty aimed at promoting healthy eating.			x		x
23.	A pharmaceutical company that recently developed nutritional supplements wants to fund an			x		x

	SCENARIOS	Size of grant in USD (< 50,000; 50-99 K; 100-249 K; 250-499 K; 500-999 K; 1 million or more)	Type of grant (individual; research team; research center; endowed chair)	Type of for-profit organization (food and beverage; pharmaceutical; tobacco industry; petroleum; health insurance, gambling, alcohol industry)	Target population (disadvantaged communities; women; children; specific diseases or health outcomes; developing countries, arm dealing and manufacturing,)	Type of research/practice (basic science; bedside (patients/hospitals); population-based; policy; intervention)
	intervention at your faculty aimed at promoting exercise.					
24.	A billionaire, whose wealth comes primarily from telecommunications but who also has investments in tobacco companies, wants to set up a family health centre at your university to support innovative programs in maternal and child health.		**x**	x	x	x
25.	A corporation in the sports clothing industry with factories in third world countries with a questionable environmental record wants to sponsor a 'greening the environment' initiative at your university.			x	x	x
26.	A tobacco company offers you funding for a study investigating the impact of			x		x

	SCENARIOS	Size of grant in USD (< 50,000; 50-99 K; 100-249 K; 250-499 K; 500-999 K; 1 million or more)	Type of grant (individual; research team; research center; endowed chair)	Type of for-profit organization (food and beverage; pharmaceutical; tobacco industry; petroleum; health insurance, gambling, alcohol industry)	Target population (disadvantaged communities; women; children; specific diseases or health outcomes; developing countries, arm dealing and manufacturing,)	Type of research/practice (basic science; bedside (patients/hospitals); population-based; policy; intervention)
	tobacco products and e-cigarettes.					
27.	A multinational corporation that manufactures soft drinks, juices and packaged junk food is seeking to recruit Public Health researchers from your faculty in order to conduct a study on fitness and other health related topics for children.		x	x	x	x
28.	A financial services corporation establishes a foundation with its namesake but with an independent Board of Trustees. This foundation wants to sponsor fellowships in health care financing at your university.		x	x		
29.	A billionaire, whose wealth comes primarily from arms sales, wants to donate money to		x	x		

	SCENARIOS	Size of grant in USD (< 50,000; 50-99 K; 100-249 K; 250-499 K; 500-999 K; 1 million or more)	Type of grant (individual; research team; research center; endowed chair)	Type of for-profit organization (food and beverage; pharmaceutical; tobacco industry; petroleum; health insurance, gambling, alcohol industry)	Target population (disadvantaged communities; women; children; specific diseases or health outcomes; developing countries, arm dealing and manufacturing,)	Type of research/practice (basic science; bedside (patients/hospitals); population-based; policy; intervention)
	construct a building in your university with his name on it.					
30.	A pharmaceutical company that manufactures chemotherapy drugs wishes to sponsor an intervention campaign to screen for breast cancer at your university's infirmary.		x	x	x	x
31.	A pharmaceutical firm recently fought against its drugs being manufactured in India as generics, arguing patent and intellectual property rights. It wants to support research in health policy at your university.		x	x		x
32.	An alcohol industry donates money to your university's Office of Grant. The office will be responsible for the distribution and allocation of the money for various projects		x	x		

	SCENARIOS	Size of grant in USD (< 50,000; 50-99 K; 100-249 K; 250-499 K; 500-999 K; 1 million or more)	Type of grant (individual; research team; research center; endowed chair)	Type of for-profit organization (food and beverage; pharmaceutical; tobacco industry; petroleum; health insurance, gambling, alcohol industry)	Target population (disadvantaged communities; women; children; specific diseases or health outcomes; developing countries, arm dealing and manufacturing,)	Type of research/practice (basic science; bedside (patients/hospitals); population-based; policy; intervention)
	without directly acknowledging the alcohol company's involvement					
33.	An international tobacco company, in partnership with a global labour organization, wants to fund an advocacy campaign in order to stop the exploitation of child labourers in tobacco farming and approach you to plan and evaluate such a campaign.			x	x	x
34.	A warehouse department store, whose employees suffer from exploitation and violence at the workplace, wants to donate $10'000 for facilities and equipment to your faculty.	x	x	x		
35.	A fast-food corporation wants to donate $5'000 for a one-day students' health education	x	x	x		x

	SCENARIOS	Size of grant in USD (< 50,000; 50-99 K; 100-249 K; 250-499 K; 500-999 K; 1 million or more)	Type of grant (individual; research team; research center; endowed chair)	Type of for-profit organization (food and beverage; pharmaceutical; tobacco industry; petroleum; health insurance, gambling, alcohol industry)	Target population (disadvantaged communities; women; children; specific diseases or health outcomes; developing countries, arm dealing and manufacturing,)	Type of research/practice (basic science; bedside (patients/hospitals); population-based; policy; intervention)
	activity organized by your Public Health School.					
36.	An international businessman who manages global investments in oil, gas wants to donate $20 million to the renovation and expansion of your medical Center at the university.	x	x	x		
37.	A company that manufactures fertilizers and pesticides wants to sponsor a research study your faculty is conducting on farmers' protective clothing.			x	x	x
38.	A recognized foundation recently divested from its tobacco stocks, wants to fund a smoking cessation program that is being implemented at your university.			x		x
39.	A multinational phone company wants to donate	x		x	x	x

	SCENARIOS	Size of grant in USD (< 50,000; 50-99 K; 100-249 K; 250-499 K; 500-999 K; 1 million or more)	Type of grant (individual; research team; research center; endowed chair)	Type of for-profit organization (food and beverage; pharmaceutical; tobacco industry; petroleum; health insurance, gambling, alcohol industry)	Target population (disadvantaged communities; women; children; specific diseases or health outcomes; developing countries, arm dealing and manufacturing,)	Type of research/practice (basic science; bedside (patients/hospitals); population-based; policy; intervention)
	$500’000 to support a project for working children that your faculty is conducting. This company has recently been in the news for exploiting their workers					
40.	A gambling company wants to donate $1.2 million to your university’s art and music department. The donation will go toward an initiative the arts/music department is working on to build a visual and performing arts centre for the youth in an impoverished neighbourhood of the university	x	x	x	x	
